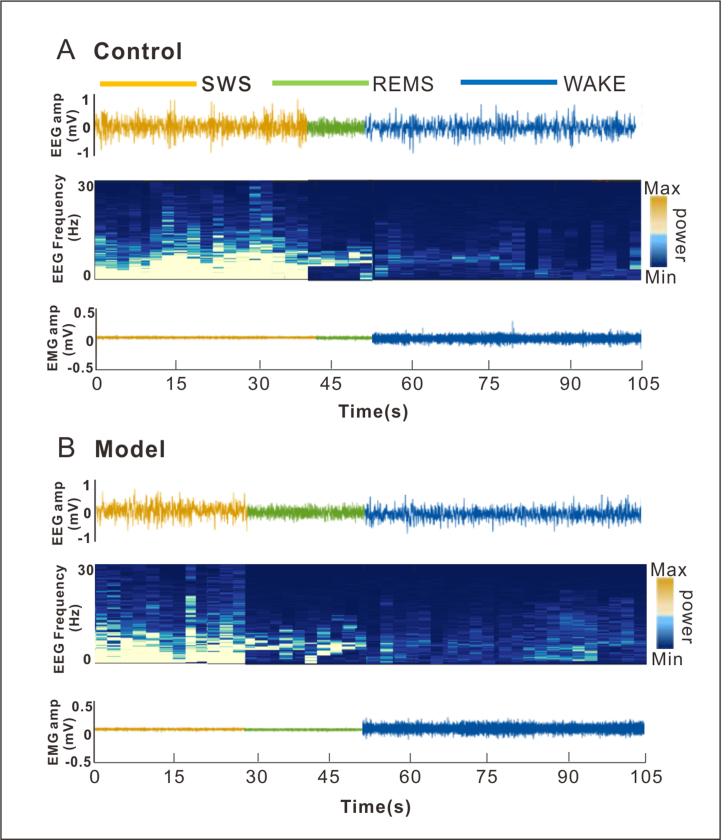# Establishment of insomnia model of chronic unpredictable stress in rats

**DOI:** 10.1016/j.heliyon.2023.e18338

**Published:** 2023-07-17

**Authors:** Wenhui Zhang, Xingping Zhang, Deqi Yan, Guanying Wang, Qingquan Wang, Xiaojuan Ren, Tao Liu

**Affiliations:** a Xinjiang Medical University, The Fourth Clinical College of Xinjiang Medical University, China; b College of Traditional Chinese Medicine, Guizhou University of Traditional Chinese Medicine, China; c Henan Provincial Hospital of Traditional Chinese Medicine, China; d Xinjiang Medical University Affiliated to Urumqi Hospital of Traditional Chinese Medicine, China

**Keywords:** Insomnia model, Stress, Emotion, Memory

## Abstract

It is well known that stressful situation is one of the important factors causing insomnia, however, the underlying mechanism is still elusive. Therefore, the establishment of a suitable animal model of stress insomnia will be of great help to solve this problem. In this study, by combining with chronic unpredictable stress (multitude of stressors) and sleep deprivation, we attempted to establish a rat model of stress insomnia. It was observed that rats with stress insomnia showed significant weight loss, and less sleep quality in pentobarbital sodium induced sleep test and electroencephalogram detection. Moreover, rats with stress insomnia showed greater depression and anxiety detected by forced swimming, sucrose preference test and open field. Since oxidative stress has been reported to be involved in insomnia, we further evaluated the production of oxidative stress and found that the levels of lipid peroxidation product malondialdehyde (MDA) in liver, serum total bilirubin and urine biopyrrin were all significantly increased in rats with stress insomnia. In addition, we also found that the memory of these rats with stress insomnia was also obviously reduced in water maze. Taken together, these results demonstrate that the emotional behaviors, memory, oxidative and metabolism of the rats were all significantly changed after modeling, indicating a rat model of stress insomnia was successful establishment, and this animal model will provide basis to further explore the underlying mechanism of chronic stress in insomnia.

## 1 Introduction

Insomnia and stress are strongly associated and pathophysiologically integrated [[1], [2], [3]]. The occurrence of stress increases the risk of insomnia, and if the psychological pressure is not timely elimination or treatment, stress insomnia will be transformed into chronic insomnia [1,4]. It has been reported that the estimated prevalence of insomnia symptoms in the general adult population is 30%–50% [5]. As a worldwide public health problem, chronic insomnia has received more and more attention. It not only affects the daytime function and quality of life of patients, but also increases the incidence of cardiovascular disease, diabetes, mental disease and other sleep disorders, causing a huge burden to individuals and society [6]. Although many experts have created many pathogenesis models for insomnia disorders from the perspectives of biology, behavior and psychosocial factors [[7], [8], [9], [10]], the pathogenesis and etiology of chronic stress insomnia disorders have not been fully elucidated.

It's known that humans and experimental animals share the same homeostasis and circadian mechanisms for sleep and the animal models are always to test experimental hypotheses and clinical hypotheses to better understand the occurrence and development of human diseases and study prevention and treatment measures [11,12]. Therefore, the use of animal models has become an extremely important experimental means in modern biomedical research. Hypnotic drug research is no exception and its related insomnia animal model has become the main carrier of hypnotic drug evaluation. Insomnia is not only an important manifestation of depression [13], anxiety [14] and other neurological diseases [15,16], the consequences of long-term insomnia is often aggravating the symptoms of the disease, so that the patient falls into a vicious circle of insomnia, depression, anxiety and insomnia [14,17]. Insomnia will also reduce the body immunity and induce endocrine disorders [18]. The sleep characteristics of rodents such as rats and mice are naturally different from those of humans [19], so the recent animal models can only partially simulate the characteristics of human insomnia. However, there are many similarities between human and rodent sleep in terms of homeostasis regulation, sensitivity to endogenous and environmental factors, and response to wakefulness and hypnotic drugs, which are the premise of using animal models for insomnia research [20].

Previous studies have shown that oxidative stress plays an important role in the occurrence and development of many diseases, including diabetes, cardiovascular disease, depression and sleep apnea syndrome [[21], [22], [23]]. The results of some animal studies showed that the serum malondialdehyde (MDA) level increased and glutathione peroxidase (GSH-Px) level decreased in sleep-deprived rats [24]. It suggests that sleep disorders may be related to oxidative stress. However, there is still a lack of research on the relationship between liver MDA in patients with chronic insomnia. Therefore, by measuring the changes of oxidative stress markers in patients with insomnia, including liver MDA, as well as the possible influencing factors and clinical correlation, we aimed to explore the relationship between oxidative stress and insomnia.

In addition, bilirubin is an essential antioxidant, it is known that the oxidative metabolite of bilirubin, biopyranone, is a sensitive marker of oxidative stress in urine [25]. Biopyrrin is the final product of the oxidation reaction between bilirubin and reactive oxygen species. During oxidative stress, bilirubin is converted into biopyrrin and excreted in urine [26]. In view of this, in recent years, attention has been paid to the problem of how to use urine excretion as an indicator of oxidative stress in life. It is known that surgery, emotional stress, endotoxin administration, and ischemia reperfusion can lead to increased biopyrrin [[27], [28], [29], [30]]. Since stress is closely related to insomnia, whether biopyrrin can be used as an indicator for the detection of insomnia induced by chronic unpredictable stress remains to be further determined.

In this study, we attempted to establish a rat model of stress insomnia by combining with chronic unpredictable stress (multitude of stressors) and sleep deprivation. We detected the emotional behavior, learning ability, oxidative and metabolism of these model rats, hoping to provide experimental evidence for further research on insomnia induced by chronic unpredictable stress.

## 2 Material/methods

### 2.1 Animals

Sprague-Dawley male rats (2-month-old, 180–220 g) used in this study were provided by Animal Experimental Center of Xinjiang Medical University, License No. SCXK (new) 2018-0002. The rats were kept in the Animal Experiment Center of Xinjiang Medical University (SPF grade). In this study, we formulated the zeitgeber time (ZT), ZT0 is the time when the light is on (10:00) at morning, ZT12 is the time when the light is off (22:00) at night. Each day was divided into two phases: light phase (ZT0:00–12:00) and dark phase (ZT12:00–24:00). The temperature of the feeding room was 23°C–25 °C, the humidity was 30%–60%. The rats had free access to water and food. Their health and behaviors were monitored daily. After 7 days of acclimation in the Animal Experiment Center of Xinjiang Medical University, rats were randomly divided into model group and blank group. Unless otherwise stated, all behavioral experiments were performed during the light phase of the cycle. Experimental procedures were conducted in accordance with the China Experimental Animals Administration Legislation and were approved by the Ethics Committee of Xinjiang Medical University (Ethical Approval number: IACUC-20210115-07).

### 2.2 Rat model

To establish the insomnia model of chronic unpredictable stress in rats, experiment was carried out as described previously [31]. Briefly, during the entire 28-day molding process, the male rats in model group received randomly the following eight stimuli during the light phase: ① The rat tail was gently clamped for 3 min; ② crowded for 3 h; ③ swimming in cold water at 4 °C for 5min; ④ swim in warm water at 45 °C for 5min; ⑤ the foot shock for 10 s (0.6 mA); ⑥ shake the cage horizontally for 10 min; ⑦ fasting for solids and liquids; ⑧ wet wood chips for 2 h. Besides, the male rats in model group received the following six stimuli during the dark phase: ① damp sawdust; ② a fast; ③ light (300 lux); ④ loneliness; ⑤ tilting squirrel cage; ⑥ fasting for solids and liquids. Moreover, no more than 5 times of each stimulus during the light phase and no more than 6 times during the dark phase, so that the rats could not anticipate the occurrence of the stimulus. In addition, starting from the 15th day, rats were subjected to an additional intermittent restraint in a transparent tube with a diameter of 6.2 cm and a volume of 750 ml for a total of 8 h per day for 14 consecutive days, and during this period, the room was continuously subjected to noise of more than 70 dB and less than 80 dB. Rats in control group were fed normally during the entire 28-day process. For collecting the rat urine, the rat urine metabolism tank was used to collect the rat urine at night (single cage, fasting, unable to restrain water). The urine was collected in a sterile tube and centrifuged at 2500 rpm at 2–8 °C for 20 min. The supernatant urine was collected. For collecting the serum of rats on day 29, rats were anesthetized by intraperitoneal injection of zolazepam hydrochloride (10 mg/kg, Zoletil 50, BN 7UX8A, Virbac S. A. Co. LTD) and Serrazine hydrochloride (10 mg/kg, 2020110, Dunhua Shengda Animal Medicine Co. LTD), and then blood samples were taken from the abdominal aorta (4 ml/rat). The serum was obtained by centrifuging the collected blood at 2000 rpm for 15min. In addition, for collecting the liver tissues, the anesthetized rats were quickly moved to ice for dissection and the liver tissues were removed and collected. All the samples were partitioned and labeled in time and stored at −80 °C.

### 2.3 Forced swimming test

The experiment was carried out as described previously [32]. Transparent cylindrical transparent container was used for forced swimming experiment, with a bottom diameter of 20 cm, a height of 50 cm, a water temperature of 23 °C and a water depth of 30 cm. The experiment was divided into two days. On the first day, rats were placed in a forced swimming bucket filled with water, and the animals could swim in the bucket for 15 min. On the second day, each rat was put into the bucket again and observed for 6 min, and the total immobile time within 4 min was counted. The criterion for immovability was to float vertically in the water, while performing only those movements necessary to keep the head above water. At the end of the experiment, the rats were removed and dried with a towel.

### 2.4 Pentobarbital sodium sleep test

The protocol of pentobarbital sodium sleep test likes those described previously [33]. Briefly, The rats were fasted from ZT3:00 on the 37th day of modeling, and the pentobarbital sodium sleep test was conducted 24 h later at ZT3:00–7:00 on the 38th day. On the day of test, rats were intraperitoneally injected with pentobarbital sodium (35 mg/kg, CAS: 9060-05-3, Sigma), and then the sleep latency and sleep time of the rats were recorded. Sleep latency refers to the period from the end of pentobarbital sodium injection to the disappearance of righting reflex for 60 s in rats. Sleep time refers to the period from the end of the sleep incubation period to the recovery of spontaneous activity in rats.

### 2.5 Electroencephalogram

Electroencephalogram (EEG) was performed according the previous description [34]. In brief, after the rats were anesthetized by intraperitoneal injection of sodium pentobarbital (40 mg/kg), the head of the rats was fixed and positioned with a brain stereoscopic locator, and EEG recording electrode screws were installed. 4 electrode screws were installed with 2 screws fixed 2 mm in front of the coronal suture and 2 screws fixed 2 mm in front of the lambdoidal suture. The screws were either 5 mm left or right to the sagittal suture. Each screw was welded to a 4 mm silver wire to match the corresponding EEG wire of the 6-wire micro socket. Besides, there are also two sterile wires for electromyogram (EMG) recording were directly inserted into the head and back neck muscles. After the rats woke up, they were placed in a single cage. EEG and EMG signals were recorded on day 4 postoperatively using the rat EEG detection system (Pinnacle, USA model: 8200-K2/4-SL), and AccuSleep software is applied for off-line analysis in MATLAB (MathWorks, Natick, MA, USA) according to the mentioned standard below to determine the behavior state for 24 h. At the same time, infrared night vision camera (Shenzhen Pulian Technology Co., LTD., Model: TL-LPC43AN-4) and TP-LINK security system (×64) (Shenzhen Pulian Technology Co., LTD., version: 2.12.17.248) were used for synchronous video recording. Then, the sleep/awakening cycle of rats is divided into three phases: awakening, slow-wave sleep (SWS) and rapid eye movement sleep (REMS). The duration of any independent sleep phase shall be at least 5s, and every 5s shall be taken as an analysis unit.

Awakening: The awakening is characterized by low-amplitude fast theta (6–9 Hz) waves. At this stage, the rats could be in different behavioral states: walking, climbing, searching, eating, drinking, wiping face, licking body, or standing still.

SWS: At the beginning of SWS phase, the EEG of rats gradually showed relatively short fusiform waves (10–15 Hz) and delta waves (0.5–5 Hz). EMG showed low muscle tone at this stage. At this stage, rats lie still and close their eyes.

REMS: The cortical EEG is dominated by low amplitude and high frequency signal theta waves in REMS phase, which is not significantly different from the awakening phase, and the EMG shows low muscle tone in this phase. During this phase, the rats were in a reclining sleep state. Since the awakening phase cannot directly enter the REMS sleep phase, there is always SWS phase before the REMS phase, and REMS can directly turn into awakening phase or SWS phase.

### 2.6 Open field test

To observe the autonomous behavior, exploratory behavior and stress of experimental animals in new environment, the open field test was used [35,36]. Although rodent animals are afraid of the new open environment, and they mainly move in the surrounding area, but less in the central area, the exploratory characteristics of the animals make them move in the central area. Therefore, the anxiety caused by this can also be observed. Open field test was carried out in the open field box, the open field box was 100 cm (length) × 100 cm (width) × 40 cm(height), the bottom of the box was 100 cm^2^, the bottom and the periphery of the box were black, the bottom was divided into 25 squares (20 cm × 20 cm) on average, and the activities of rats within 5 min were recorded by video. The peripheral area is divided into 1 area, and the sub-central area and the central area are divided into 2 areas. Rats were placed in the center of the bottom of the box at the beginning, and counted the number of horizontal movements on the bottom of the box (counted as one time when both front PAWS crossed one box at the same time), and the number of horizontal movements was the number of boxes the rats crawled through in 5 min (horizontal score). At the end of each experiment, the rats were taken out, and the test box was cleaned and sprayed with 20% ethanol to mask the odor of rat left in the test box.

### 2.7 Sucrose preference test

The experiment was carried out as described previously [37]. 1) Each rat was reared in a single cage, and food was provided as usual. At the same time, a bottle of 1% sugar water and a bottle of water were placed in each cage, with a volume of about 100 ml. The position of the two bottles should be changed every 12 h to avoid positioning bias in rats. 2) After 3 days, the food and water were removed, and the rats were deprived of food and water for 24 h. 3) The weight of bottles containing sugar water and water was weighed in advance, and a bottle of 1% sucrose water and water was placed in each cage respectively. After 1 h, the consumption of sugar water and water was recorded. At this time, the rats were provided with food, and the consumption of sugar water and water was recorded again 24 h later. 4) The sucrose preference index of all the rats was evaluated, the sucrose preference index was expressed as (Δweight sucrose)/(Δweight sucrose + Δweight water) × 100%.

### 2.8 Morris water maze

The Morris water maze was carried out as described previously [38], and it consists of a grey board circular pool, 120 cm in diameter and 50 cm high; The water depth in the pool is 30 cm; White food dye was added into water to hide platforms; The water temperature was kept at (22 ± 2)°C. Different reference objects were set outside the maze as the reference for rats to distinguish spatial direction, and their positions remained unchanged during the experiment. The pool is divided into four quadrants NE, NW, SE and SW by a diagonal across the center of the pool. The water entry point is set at the central point of each quadrant near the pool wall. The platform is 10 cm in diameter and freely adjustable in height, hidden about 0.5 cm underwater. A video camera with a display system is installed directly above the maze, and the water maze data acquisition and analysis software (American CoulBourn Instrument) records relevant data and image results. During the training, the platform was underwater and was fixed in the center of the SW quadrant. At the entry point, the animal is placed in the water facing the pool wall, and the time required for the animal to search for and climb onto the platform is recorded, i.e., escape latency. Each rat entered the water in a random order, but all animals entered the water in the same order. If the animal did not find the platform within 60 s, the experimenter dragged the animal to the platform, and the escape incubation period was recorded as 60 s. The animal stays on the platform for 20 s. Dry the animal quickly at the end of each training session to keep it warm. The average latency of escape was calculated for each group four times a day to reflect the spatial reference learning ability of animals. The space exploration test was conducted a day later. After the platform was removed, each rat explored freely for 120 s. The total distance and time spent by the animals in the target quadrant and the number of times the animals entered the area were recorded.

### 2.9 Enzyme-linked immunosorbent assay (ELISA)

Malondialdehyde (MDA) and total bilirubin (TB) were extracted from liver and serum and detected by ELISA (No. ml077384 for MDA, No. ml307098 for TB, Shanghai Enzyme-linked Biotechnology Co., Ltd., Shanghai, China) according to the kit instructions. Biopyrrin was extracted from urine and detected by ELISA (No. ml407719-2 for Biopyrrin, Shanghai Enzyme-linked Biotechnology Co., Ltd., Shanghai, China), either. Briefly, the samples, standard samples and HRP-labeled detection antibodies were added into the coated micropores precoated with malondialdehyde (MDA), total bilirubin (TB) or biopyrrin antibodies, then incubated and washed thoroughly. The substrate TMB, catalyzed by peroxidase, is used to produce color. TMB is converted to blue by the action of acid to the final yellow color. The depth of the color was positively correlated with the malondialdehyde (MDA), total bilirubin (TB) or biopyrrin in the sample. The absorbance (OD) was measured at 450 nm with a microplate analyzer, and the concentration of the sample was calculated.

### 2.10 Statistics

Data are presented as mean ± SEM. SPSS software (version 21.0, IBM Corporation) was used for statistical analysis. Student's t-test was used to compare the differences between the two groups. For the one-factor analysis of variance, one-way analysis of variance (ANOVA) was used followed by Tukey's post-hoc test to compare the differences among multiple groups. For the two-factor analysis of variance, two-way ANOVA was used. *P* < 0.05 was considered a statistically significant difference.

## 4 Results

### 4.1 The body weight and sleep of rats with stress insomnia

As shown in Fig. 1A, after treatment with the chronic unpredictable stress (multitude of stressors) and sleep deprivation in rats, we observed that the body weight was reduced significantly in the group of rats with stress insomnia compared with the group of control rats (Fig. 1B and supplementary Fig.1, *p* < 0.001). Moreover, after each group was intraperitoneally injected with pentobarbital sodium, the sleep latency and sleep time of the rats were recorded. It was found that the sleep time was also reduced (Fig. 1C, *p* < 0.01), while the sleep latency was increased (Fig. 1D, *p* < 0.001) in the group of rats with stress insomnia compared with the group of control rats. In addition, the EEG results showed the total sleep time (supplementary Fig.2 and Fig. 1E, *p* < 0.05) and slow-wave sleep time (supplementary Fig.2 and Fig. 1F, *p* < 0.05) were significantly reduced in model rats compared with control rats. However, the rapid eye movement time was significantly increased in model rats compared with control rats (supplementary Fig.2 and Fig. 1G, *p* < 0.01). Taken together, these results suggest that the treatment with chronic unpredictable stress and sleep deprivation affects the weight gain and sleep of rats.

**Fig. 1:**
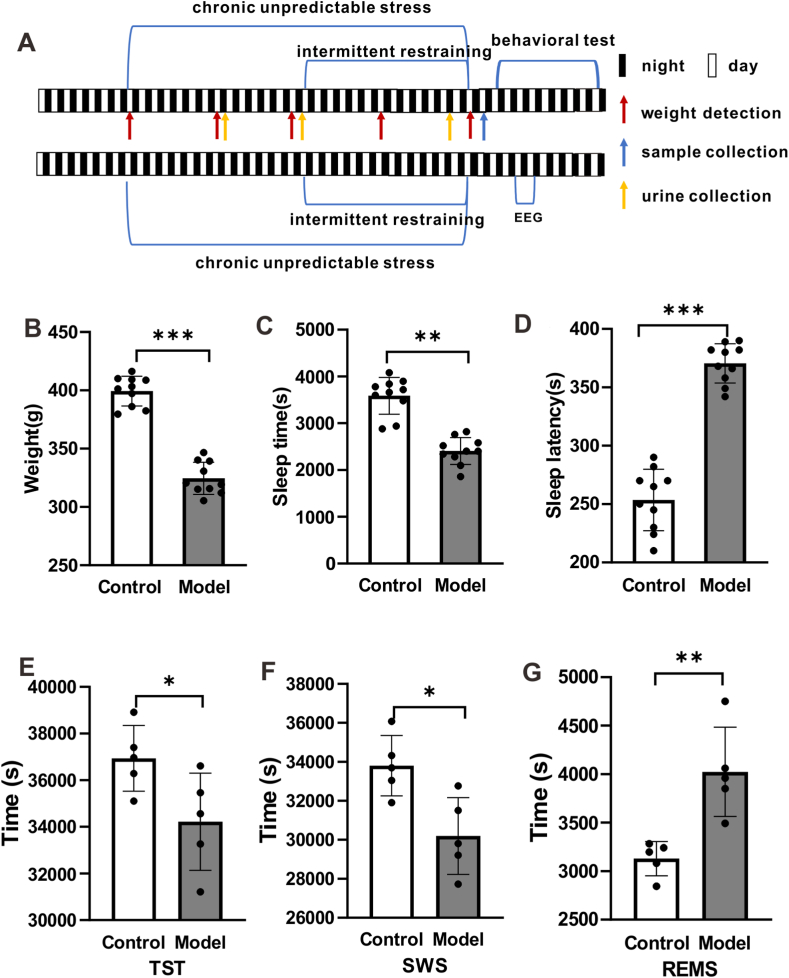
**The body weight and sleep of rats with stress insomnia.** (A) Schematic of behavioral procedure for the construction of stress insomnia model. n = 10 rats. (B) The treatment of chronic unpredictable stress (multitude of stressors) and sleep deprivation reduced the body weight of rats. n = 10 rats. (C) The treatment of chronic unpredictable stress (multitude of stressors) and sleep deprivation reduced the sleep time of rats. n = 10 rats. (D) The treatment of chronic unpredictable stress (multitude of stressors) and sleep deprivation increased the sleep latency of rats. n = 5 rats. (E) The total time detected in EEG. (F) The slow-wave sleep time detected in EEG. n = 5 rats. (G) The rapid eye movement time detected in EEG. n = 5 rats. Data are presented as mean ± SEM and analyzed by Student's t-test.*p < *0.05,* **p < 0.01, ***p < 0.001.

### 4.2 The emotion of rats with stress insomnia

**Fig. 2:**
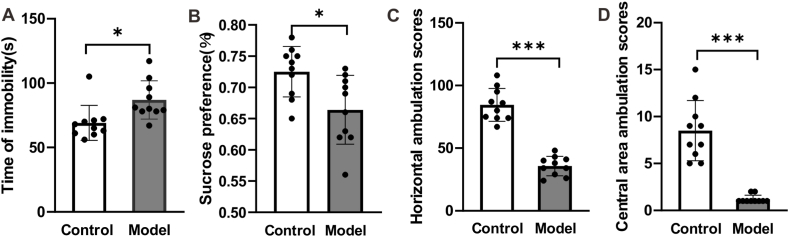
**Chronic unpredictable stress affects the depression and anxiety of rats.** (A) The treatment of chronic unpredictable stress (multitude of stressors) and sleep deprivation increased the immobility time of rats in forced swimming test. (B) The treatment of chronic unpredictable stress (multitude of stressors) and sleep deprivation reduced the sucrose preference of rats in sucrose preference test. (C) The treatment of chronic unpredictable stress (multitude of stressors) and sleep deprivation reduced the horizontal ambulation scores and central area ambulation scores of rats. Data are presented as mean ± SEM and analyzed by Student's t-test. *p < 0.05, ***p < *0.001,* n = 10 *rats.*

Then, to investigate whether the chronic unpredictable stress (multitude of stressors) and sleep deprivation used in our study established the insomnia model in rats, we firstly detected the anxiety and depression of rats since anxiety and depression is the mainly manifestation of insomnia. As shown in Fig. 2, the immobility time in forcing swimming increased significantly (Fig. 2A, *p* < 0.05), while the sucrose preference reduced significantly in the group of model rats compared with the group of control rats (Fig. 2B, *p* < 0.05). Moreover, both horizontal and central area ambulation scores decreased significantly in the group of model rats compared with the group of control rats (Fig. 2C and D, *p* < 0.001). Taken together, these results suggest that rats treated with the chronic unpredictable stress (multitude of stressors) and sleep deprivation exhibited anxiety and depression.

### 4.3 The oxidative stress of rats with stress insomnia

**Fig. 3:**
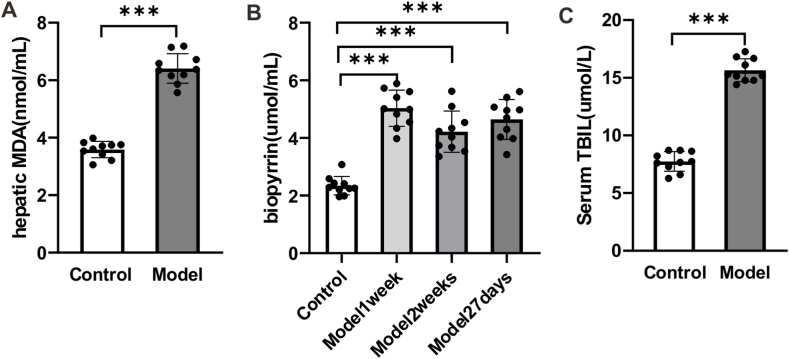
**Chronic unpredictable stress has effect on the oxidative stress of rats.** (A) The treatment of chronic unpredictable stress (multitude of stressors) and sleep deprivation increased the content of hepatic MDA in rats. Data are presented as mean ± SEM and analyzed by Student's t-test. (B) The treatment of chronic unpredictable stress (multitude of stressors) and sleep deprivation increased the content of biopyrrin in rats. Data are presented as mean ± SEM and analyzed by one-way analysis of variance (ANOVA) and Tukey test. (C) The treatment of chronic unpredictable stress (multitude of stressors) and sleep deprivation increased the content of serum total bilirubin of rats. Data are presented as mean ± SEM and analyzed by Student's t-test. ***p < *0.001,* n = 10 rats.

To further investigate the oxidative stress index of rats treated with chronic unpredictable stress (multitude of stressors) and sleep deprivation, we evaluated the content of liver MDA, serum total bilirubin and urine biopyrrin of rats. The results showed that the content of MDA was significantly increased in the group of model rats compared with the group of control rats (Fig. 3A, *p* < 0.001). Meanwhile, both the content of serum total bilirubin and urine biopyrrin were also increased in the group of model rats compared with the group of control rats (Fig. 3B and C, *p* < 0.001). Altogether, these results suggest that rats treated with the chronic unpredictable stress (multitude of stressors) and sleep deprivation exhibited enhanced oxidative stress.

### 4.4 The cognitive function of rats with stress insomnia

**Fig. 4:**
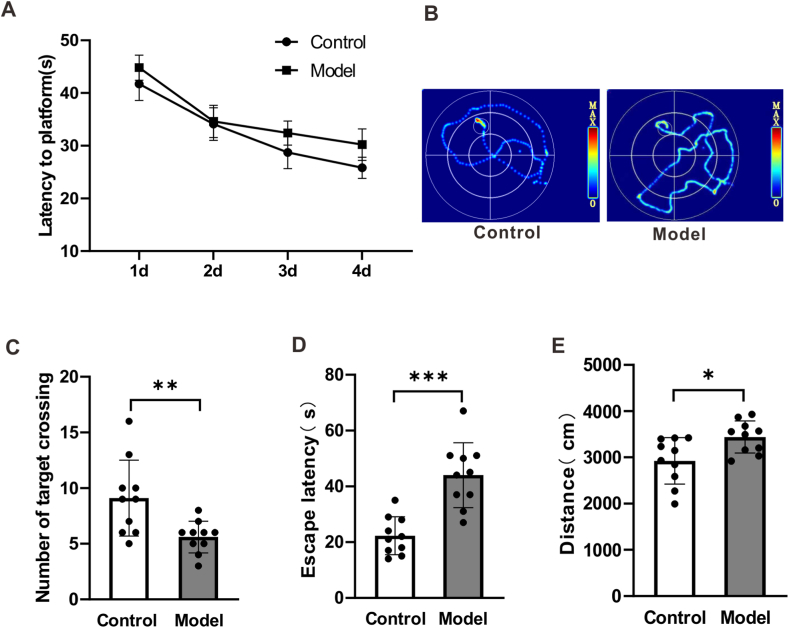
**Chronic unpredictable stress impaired the memory of rats.** (A) The treatment of chronic unpredictable stress (multitude of stressors) and sleep deprivation had no effect on the memory acquisition in water maze. Data are presented as mean ± SEM and analyzed by two-way analysis of variance (ANOVA). (B) Trajectory of rats to arrive at target platform at the fourth day monitored by thermal imaging technique. (C) The treatment of chronic unpredictable stress (multitude of stressors) and sleep deprivation decreased the number of targets crossing of rats. Data are presented as mean ± SEM and analyzed by Student's t-test. (D) The treatment of chronic unpredictable stress (multitude of stressors) and sleep deprivation increased the escape latency of rats. (E) The treatment of chronic unpredictable stress (multitude of stressors) and sleep deprivation decreased the move distance of rats during the exploration. Data are presented as mean ± SEM and analyzed by Student's t-test. *p < 0.05, **p < 0.01, ***p < *0.001*, n = 10 rats.

To further evaluate the cognitive function of rats treated with chronic unpredictable stress, we detected the memory of rats in water maze. It was found that there was no difference in memory acquisition between the group of model rats and the group of control rats (Fig. 4A and B, *p* > 0.05). However, on the memory retrieval phase, it was found that the number of target crossing was significantly decreased in the group of model rats compared with the group of control rats (Fig. 4C, *p* < 0.01). Moreover, the escape latency and total distance were found to be increased in the group of model rats compared with the group of control rats (Fig. 4D and E, *p* < 0.001, *p* < 0.05). Altogether, these results suggest that rats treated with the chronic unpredictable stress (multitude of stressors) and sleep deprivation impaired the memory of rats.

## 5 Discussion

Sleep disorder is a public health problem that should be paid attention to be solved. In addition to clinical studies, basic experimental studies are most used to study human sleep problems, and the establishment of an appropriate insomnia model is the premise to determine whether the experiment can be successful. In this study, we constructed an insomnia model in rats by combining with chronic unpredictable stress (multitude of stressors) and sleep deprivation, and found that the insomnia model rats not only exhibited sleep disorder, but also exhibited anxiety and depression, enhanced oxidative stress and impaired memory, suggesting that an insomnia model of rats was successfully induced.

At present, the commonly used sleep deprivation methods for rodents in basic research mainly include horizontal stage deprivation, stress deprivation, chemical reagent deprivation, gentle stimulation deprivation and forced exercise deprivation, etc [39]. However, there are still many areas to be improved in the development of animal models of sleep deprivation. For example, the performance of animal models of sleep deprivation is not consistent enough with the performance of human insomnia. Standardization of animal models of sleep deprivation is a challenge. In addition to the total length of sleep, the frequency of each sleep phase, the maintenance time of each sleep phase, the heart rate, blood pressure and respiratory rate of experimental animals should be paid attention to when establishing the animal model of sleep deprivation, while the changes of these indicators of insomnia in experimental animals cannot be standardized [40]. In recent years, more and more studies show that chronic stress is closely related to insomnia [41]. Especially, under the COVID-19 epidemic, the incidence and severity of epidemics related stress reactions were increased in the general population, including anxiety, depression and insomnia [[42], [43], [44]]. Studies have showed that stress responses are closely related to the aggravation of mood and insomnia [45,46]. In this study, to investigate the etiology of insomnia induced by chronic stress, we constructed the insomnia model of chronic unpredictable stress (multitude of stressors) and sleep deprivation in rats. Our results showed that the treatment of chronic unpredictable stress (multitude of stressors) and sleep deprivation not only reduced the body weight and sleep of rats, but also induced depression and anxiety of rats. These results were consistent with the previous finding from other insomnia model, such as horizontal stage deprivation and forced exercise deprivation [47,48]. Of course, each sleep deprivation method has its own key points, such as the widely used horizontal stage deprivation method, which can deprive REMS specifically. However, if the modeling time is too long or the intensity is too high, the animal may fall into the water and die because of physical exhaustion and cannot return to the platform. However, the insomnia model provided in this study is not only safe in the modeling process, but also more consistent with the performance of insomnia under the current social pressure. Moreover, this insomnia model also shows impairment of cognitive function, which provides a better model basis for the subsequent study of insomnia related to cognitive impairment.

It's known that patients always showed the performance of emotional changes or mental stimulation with insomnia as the main disease, [49,50]. Traditional Chinese medicine regards as such performance is closely related to the function of the liver [[51], [52], [53]]. In addition, more and more studies showed that oxidative stress refers to a state of imbalance between the reactive oxygen species (ROS) and the antioxidant system in the human body [22]. The cause may be excessive production of ROS or reduced antioxidant function, or both, which leads to damage of cells or tissues. It can also cause lipid peroxidation and DNA damage in the body [54]. In clinical studies, the oxidation of free radicals in human body has the characteristics of short biological half-life, low concentration, and low activity. Therefore, the degree of oxidative stress damage can often indirectly reflect the oxidative stress of human metabolites through detection. For example, the degree of lipid peroxidation in human body can be reflected by detection of MDA. In this study, we found that the content of MDA in liver, serum total bilirubin and urine biopyrrin were significantly increased in rats treated by chronic unpredictable stress (multitude of stressors) and sleep deprivation, suggesting that the elevated level of liver oxidative stress may be one of the causes of insomnia caused by chronic unpredictable stress (multitude of stressors) and sleep deprivation in rats. Of course, insomnia is the result of the comprehensive action of many factors, its mechanism involves the social environment, personal constitution and even organs, cells, molecules, and other levels. To make hypnotic drug research results more convincing, it is no longer satisfied with single factor model making to establish a hierarchical, systematic, comprehensive animal model making system of many factors will be the future development trend. At the same time, with the fusion of different experimental methods and the invention and application of various new technologies, the establishment of new animal models will be further promoted. In addition, only adult male rats were used in this study, which is also a limitation of this study. In the subsequent research, we will introduce the study of female animals and young animals, systematically evaluate the existing various modeling methods, and improve and optimize them, which can improve the accuracy, effectiveness, and reproducibility of model preparation experiments, to provide a reliable animal model for the related research on stress insomnia. Finally, attention should also be paid to sleep fragmentation in the construction of insomnia model, although we did not detect it in this work, and we will focus on it in the future. In conclusion, we constructed an insomnia model of rats by treatment with chronic unpredictable stress (multitude of stressors) and sleep deprivation, and found that this insomnia model of rats not only had difficulty sleeping, but also exhibited anxiety and depression, enhanced oxidative stress and impaired memory, suggesting that an insomnia model of rats was successfully induced. This will provide a model basis for the study of insomnia mechanism and hypnotic drugs.

Wenhui Zhang: Conceived and designed the experiments; Performed the experiments; Analyzed and interpreted the data; Contributed reagents, materials, analysis tools or data; Wrote the paper.

Xingping Zhang: Conceived and designed the experiments; Analyzed and interpreted the data; Contributed reagents, materials, analysis tools or data.

Deqi Yan, Guanying Wang, Xiaojuan Ren: Performed the experiments; Analyzed and interpreted the data.

Qingquan Wang: Conceived and designed the experiments; Performed the experiments; Analyzed and interpreted the data.

Tao Liu: Analyzed and interpreted the data.

## Data availability statement

Data included in article/supplementary material/referenced in article.

## Funding information

This work was supported by the 10.13039/501100001809National Natural Science Foundation of China [NSFC grant 81260526, 81560762, 8196150343].

## Author contribution statement

Conceived and designed the experiments: Wenhui Zhang, Xingping Zhang, Qingquan Wang.

Performed the experiments: Wenhui Zhang, Deqi Yan, Guanying Wang, Qingquan Wang, Xiaojuan Ren.

Analyzed and interpreted the data: Wenhui Zhang, Xingping Zhang, Deqi Yan, Guanying Wang, Qingquan Wang, Xiaojuan Ren, Tao Liu, Contributed reagents, materials, analysis tools or data: Wenhui Zhang, Xingping Zhang, Wrote the paper: Wenhui Zhang.

## Declaration of competing interest

The authors declare that they have no known competing financial interests or personal relationships that could have appeared to influence the work reported in this paper.

## Figures and Tables

**figs1:**
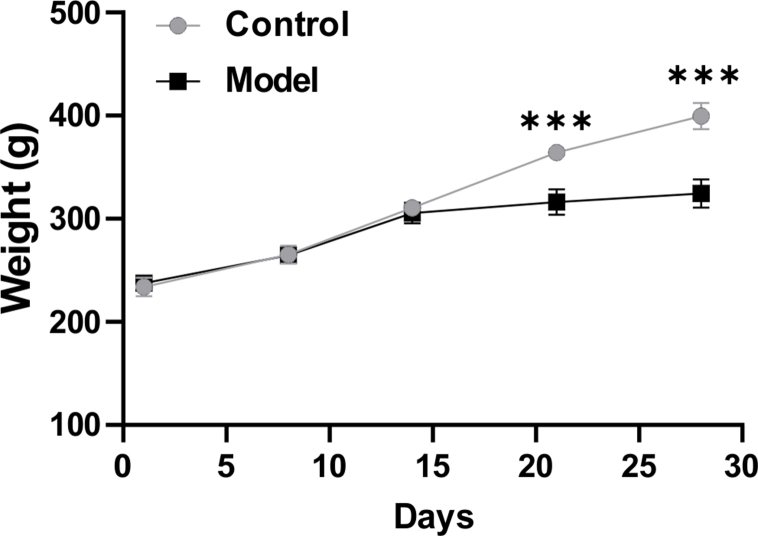


**figs2:**